# Nonclassical Crystallization and Core–Shell Structure Formation of Ibuprofen from Binary Solvent Solutions

**DOI:** 10.1021/acs.cgd.2c00971

**Published:** 2022-12-21

**Authors:** Rajaboopathi Mani, Leena Peltonen, Clare J. Strachan, Maarit Karppinen, Marjatta Louhi-Kultanen

**Affiliations:** † Department of Chemical and Metallurgical Engineering, Aalto University, FI-00076 Aalto (Espoo), Finland; ‡ Department of Physics & Nanotechnology, SRM Institute of Science & Technology, Kattankulathur 603203, Tamilnadu, India; § Drug Research Program, Division of Pharmaceutical Chemistry and Technology, University of Helsinki, 00014 Helsinki, Finland; ∥ Department of Chemistry and Materials Science, Aalto University, FI-00076 Aalto (Espoo), Finland

## Abstract

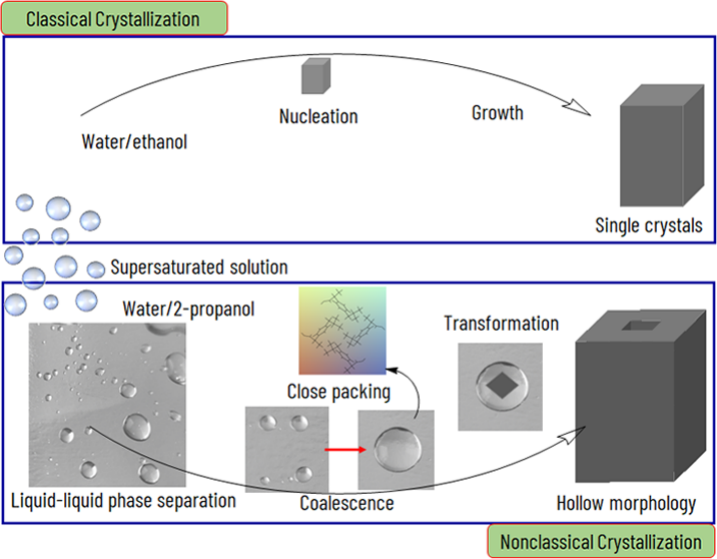

Liquid–liquidphase separation (LLPS) or dense liquid intermediates during the crystallization of pharmaceutical molecules is common; however, their role in alternative nucleation mechanisms is less understood. Herein, we report the formation of a dense liquid intermediate followed by a core–shell structure of ibuprofen crystals via nonclassical crystallization. The Raman and SAXS results of the dense phase uncover the molecular structural ordering and its role in nucleation. In addition to the dimer formation of ibuprofen, which is commonly observed in the solution phase, methyl group vibrations in the Raman spectra show intermolecular interactions similar to those in the solid phase. The SAXS data validate the cluster size differences in the supersaturated solution and dense phase. The focused-ion beam cut image shows the attachment of nanoparticles, and we proposed a possible mechanism for the transformation from the dense phase into a core–shell structure. The unstable phase or polycrystalline core and its subsequent dissolution from inside to outside or recrystallization by reversed crystal growth produces the core–shell structure. The LLPS intermediate followed by the core–shell structure and its dissolution enhancement unfold a new perspective of ibuprofen crystallization.

## Abstract 2

A liquid−liquid phase separation followed by core−shell structure formation has been observed in ibuprofen crystallization in binary solvent solutions. Liquid-phase Raman and SAXS results suggest the dense phase as an intermediate precursor phase before nucleation in nonclassical crystallization. The internal structural feature of mesocrystals enhances the dissolution.

## 1 Introduction

Crystallization is a phase transformation process from solution to solid that occurs frequently in nature and is utilized in the chemical, food, and pharmaceutical industries. Nucleation is the first step in crystallization, and the classical nucleation theory (CNT) explains the formation and growth of the nucleus.^1^ With the advancement of analytical tools,^2^ particularly in-situ experiments such as liquid-cell TEM,^3^*cryo* TEM,^4^ SAXS,^5^ and AFM^6^ to probe the crystallization directly in solution, the recent discoveries suggest that there is an alternative route (nonclassical) for the formation of crystalline solids. The nonclassical crystallization (NCC) theory explains the formation of a prenucleation phase and its transformation into the nucleus and then the crystals.^7−9^

The prenucleation phase can be a cluster or a dense phase. The theoretical study supported the two-step nucleation pathway in which the dense liquid intermediate phase appeared first and then nucleated to grow as crystals.^10^ The density functional theory (DFT) calculation was performed to further study the two-step mechanism comprising LLPS of organic small molecules.^11^ The transformation from a disordered dense liquid phase to an ordered crystalline nucleus has been reviewed by Vekilov, who concluded that the structure fluctuation occurs within the region of a higher density of molecules.^12^ The molecular interaction within the dense liquid phase and molecular structural ordering has been recently investigated for the APIs that undergo LLPS at higher pH values.^13^ The less-stable dense phase has been refuted because the molecules are kinetically stabilized by translational and rotational diffusion, which accounts for the nucleation within its metastable region. Furthermore, the binodal and spinodal limits of the corresponding liquid–liquid miscibility gap have been confirmed. The study reaffirmed the fact that the dense liquid phase of LLPS can be considered as the densified precursor phase for nucleation. Nonetheless, in the absence of a molecular-level study with crucial experimental evidence of the final product, the role of LLPS in the nucleation mechanism will be unclear as a large and visible dense phase is common in organic small molecules.^14−16^

Because the reversed crystal growth, mesocrystal formation, and oriented aggregation occur via the NCC pathway, any of these can be considered as experimental proof. The aggregation of nanoparticles, followed by surface crystallization and growth from the surface to the core are the stages of reversed crystal growth. Although reversed crystal growth has been mostly described for inorganic materials, a few studies are reported for organic materials.^17,18^ Spatially separated, self-assembled, and crystallographically oriented nanoparticles can form mesocrystals, and the spatial separation is absent in oriented crystals.^19^ To the best of our knowledge, the present study for the first time reports LLPS followed by a core–shell morphology that follows NCC in organic small molecules. We considered IBU as a model system as it undergoes LLPS during crystallization.^20^ Ibuprofen was obtained in the core–shell form (by slow evaporation of a water/2-proponal solution), as single crystals (by slow evaporation of a water/ethanol solution), and as precipitates (by cooling) (see Experimental Section S1, Supporting Information). The formation of the IBU core–shell structure via reversed crystal growth or a possible mesocrystal core with single crystals at the outside shell as an end-product of LLPS would help understand how LLPS could serve as a metastable dense precursor phase in NCC. Conducting a time-dependent study is out of the scope of this study, as the formation of a core–shell structure is well evidenced in NCC, and our prime focus is to investigate the dense phase. IBU is widely prescribed as a nonsteroidal anti-inflammatory drug, and it is a weak monoprotic carboxylic acid, but the poor aqueous solubility limits the drug potential in formulations. The crystallization kinetics and the parameters to obtain the crystals with and without LLPS as intermediates have been reported. This study considered that LLPS provides an alternative pathway to classical nucleation.^20^ A cluster of particles with a large size induced by an antisolvent was considered as the prenucleation cluster in NCC.^21^ The inherent structural features of the polycrystalline core can be strategically utilized to enhance the drug dissolution of IBU. The prerequisite dense liquid phase and molecular structural order in solution were investigated by liquid-phase Raman and SAXS. The internal cross-section of the core–shell structure was confirmed by SEM and SEM-interfaced focused ion beam (FIB) cut images. These results are used to hypothesize the underlying nucleation pathway of IBU with emphasis on the existing LLPS.

## 2 Experimental Methods

### 2.1 Materials

Racemic (R,S)-2-(4-(2-methylpropyl)-phenyl) propionic acid (IBU) was purchased from Alfa Aesar (99%) and 2-propanol from Merck (≥99.5%). Additives such as hydroxypropyl methylcellulose (HPMC), Pluromic F127, poly(vinyl pyrrolidone) (PVP), polyvinyl alcohol (PVA), and polyacrylic acid (PAA) were purchased from Merck. All chemicals were of analytical grade and were used without further purification. Milli-Q Water was used for all experiments.

### 2.2 Preparation of the Core–Shell Structure and Precipitates

**Scheme 1:**
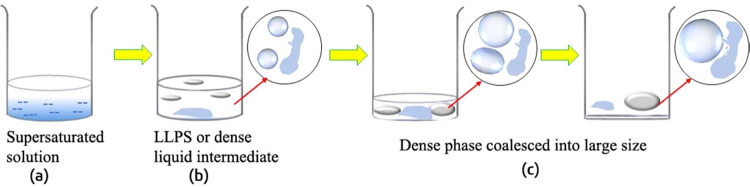
Schematic Representation of Liquid–Liquid Phase Separation (LLPS) during IBU Core–Shell Structure Formation with a Water/2-Propanol Ratio of 1.5 LLPS is a dense liquid intermediate state observed during crystallization. The dispersed solution was filtered (a), and after ca. 2 days, LLPS was occurred in the solution (b). Later, the dense liquid phases coalesced into a large drop (c, d).

Different IBU crystals were prepared by cooling, followed by evaporative crystallization (Figure S1). The solvent ratio played a decisive role in the core–shell structure formation of IBU. The core–shell structure was obtained in binary solvents of water and 2-propanol. IBU (0.6 g) was added to 10 mL of the binary mixture (0.29 M) of water and 2-propanol with a ratio of 1.5 at 50 °C. Initially, 0.5 g of IBU was dissolved slowly, and then, 0.1 g was added as a whole to obtain a dispersed solution. The different additives were added (see Section 3.2), and the final suspension was stirred for 1 h and cooled rapidly to 25 °C. No LLPS was observed during the stirring. The solution was filtered using a Whatman filter paper and kept in a controlled water bath at 25 °C for the slow evaporation of solvents. To avoid atmospheric instability, the evaporation was performed in a controlled room at a temperature of 23 °C and 50% humidity. The crystals were obtained (hereafter, IBU core–shell structure) in ca. 3 days and stored in a desiccator until used for further characterization. Interestingly, LLPS was observed after 1 day or before the nucleation, and its evolution is also further depicted in Scheme 1. The image of LLPS observed during crystallization is presented in Figure S2. It must be noted that after filtering, the solution was not very clear due to rapid cooling but turned into a clear solution after, e.g., 24 h. Then, LLPS was observed. The droplets coalesced into a larger drop with time, as shown in Scheme 1.

We could have stopped the experiment at this stage, but due to curiosity to further understand the impact of the water/2-propanol ratio, we performed the crystallization at ratios 1 and 4. By following the same experimental steps (Figure S1), for an equal mixture of 2-propanol and water, we observed a very stable dense liquid phase (ca.3 months) as an end-product. The ibuprofen solution in 2-propanol also produces a dense liquid phase. For a water/2-propanol ratio of 4, we obtained a precipitate on cooling rapidly to 25 °C. The precipitates were collected and dried in a hot air oven for further characterization (hereafter, IBU precipitate).

## 3 Results and Discussion

### 3.1 Liquid- and Solid-Phase Raman Spectra

As the molecular vibrations are sensitive to the local environment, we use Raman spectroscopy effectively to understand the structure of IBU in the solid and dense phases. DFT calculations of the inter- and intramolecular interactions of IBU with the inclusion of water and with IBU as a monomer and dimer in the gas phase were reported.^22,23^ Previously, the difference between hydrogen bonds involving dimers and extended hydrogen bonding networks in tetrolic acid has been studied.^24^ The X-ray Raman study traces the interaction of solutes in solution, particularly during nucleation.^25^

**Figure 1:**
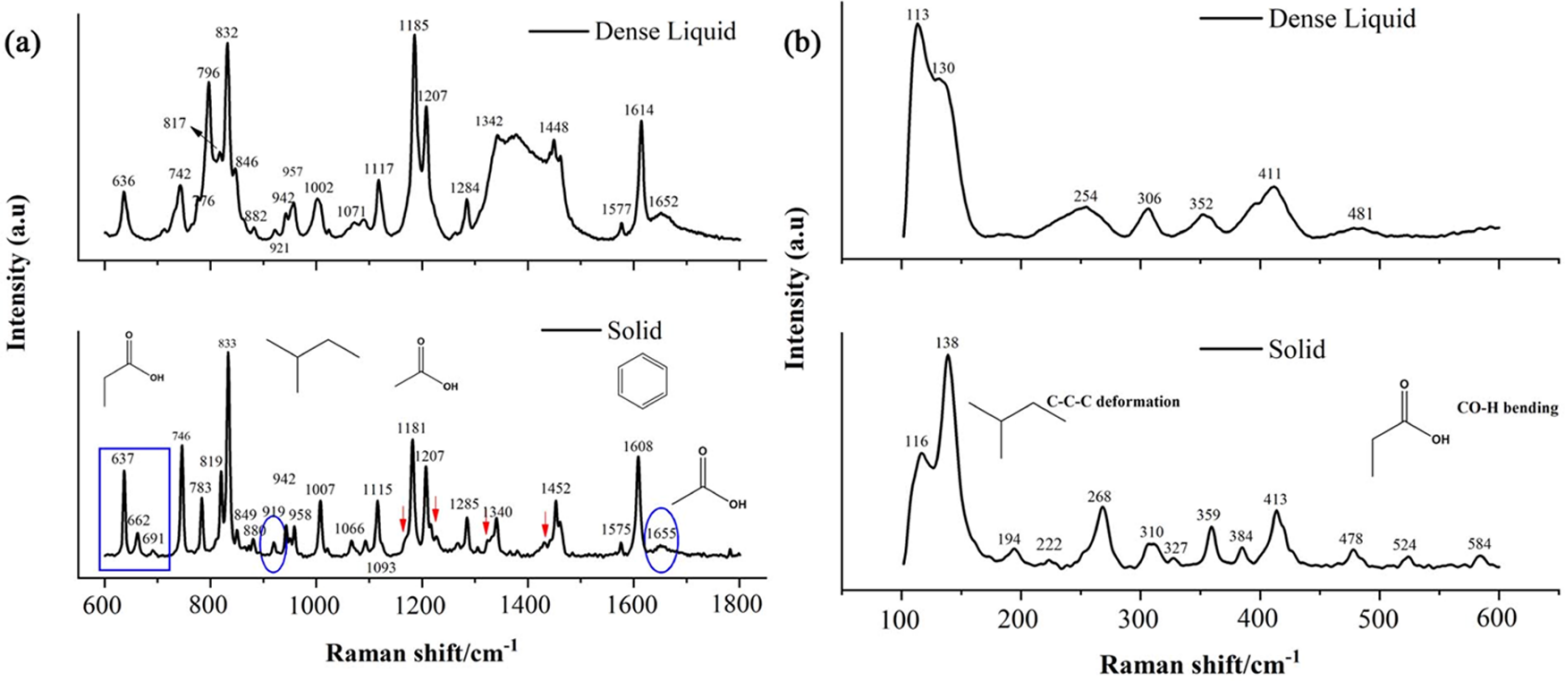
High-resolution Raman spectra of the IBU solid and the dense liquid phase in the (a) mid- and (b) low-frequency regions. In (a), red arrows indicate sensitive vibrations related to the environment. The blue ellipse at 1655 cm^–1^ indicates the carbonyl group involving hydrogen bonding and that at 942 cm^–1^ confirms the dimer formation. The C=O deformation, C–OH stretching, and alkyl vibration regions are indicated by a blue rectangle. In (b), the molecular structure of different functional groups is presented to address their corresponding vibrations in those regions. The grating used was 1800 lines/mm. The molecular structure of different functional groups is presented to represent their corresponding vibrations in those regions.

The Raman spectra in the region of 600–1800 cm^–1^ are presented in Figure 1a. This mid-frequency region mostly covers the O–H bending and C=O stretching vibrations associated with the COOH group (H-bonding). The medium to weak peaks at 637, 662, and 691 cm^–1^ for the solid are related to CO-–H out-of-plane bending, C–C=O deformation, and C–OH stretching. The corresponding peak at 636 cm^–1^ is attenuated and broadened, and the C=O deformation and C–OH stretching vibrations are completely weakened in the dense phase. The CH_3_ rocking vibration intensity was diminished in the dense phase at 742 cm^–1^. Interestingly, the C=O out-of-plane wagging and CH_3_ rocking vibrations overlapped and shifted to 796 cm^–1^ (blue shift) for the dense phase compared with that of the solid at 783 cm^–1^. These vibrations further confirmed that these changes are not only due to COOH, which is involved in the hydrogen bonding, but also due to the methyl group present in the chiral center, leading to intramolecular interactions. On another side of the benzene ring, the intensity of the CH_2_ rocking and CH_3_ vibrations of the methylpropyl group reduced for the dense phase. The line shape and intensity of the CO–H bending vibration (dimer bond) or CH_3_ rocking vibration at 942 cm^–1^ in the solid was changed compared with the dense phase (see spectra in Figure 1a). For instance, the regions 942–958 and 1066–1093 cm^–1^ are overlapped for the dense phase, which is also associated with CH_3_ and CH_2_ in the methylpropyl group. However, although this peak corresponds to the fingerprint for dimer formation, the absence of a peak shift indicates the strong dimer formation in the dense phase similar to the solid phase.^24^ There are four bending modes related to the O–H group, which are sensitive to the environment, and these vibrations are observed very weakly at 1389, 1303, 1192, and 1159 cm^–1^ (red arrow, Figure 1a) in the solids. These vibrations are absent in the dense phase, which reflects the change in the environment. Furthermore, the blue shift related to the C=O stretching at 1655 cm^–1^ was observed for the solid compared with the dense phase at 1652 cm^–1^. This carbonyl stretching is related to the carbonyl group involved in the dimerization.^24,26^ A very slight carbonyl shift (3 cm^–1^) between the solid and dense phases and also a peak at 942 cm^–1^ suggest significant dimer formation, and the absence of peaks around 1700 cm^–1^ in the dense phase indicates the absence of monomers in both phases.^27^ It is important to note that a large shift (13 cm^–1^) has been reported between the solution (not dense phase as reported here) and their solid phase for the carbonyl dimer.^28^ Conversely, a red shift has been observed for the aryl C–C stretching vibration for the solid compared with the dense phase at 1614 and 1608 cm–^1^, respectively. The adjacent vibrations at 1575 and 1577 cm^–1^ are also associated with the aryl C–C stretching vibration. These concomitant red and blue shifts clearly indicate the intermolecular interaction in both phases and the close aggregation of IBU in the dense phase. The peaks at 1575, 1608, and 1655 cm^–1^ in the solid phase further confirm the racemate formation of IBU.

The Raman spectra in the low-frequency region represent mostly the dominating aliphatic chain (C–C) and O–H bending vibrations (Figure 1b). This region strongly probes the intermolecular interaction. The homogeneous blue shift of solid samples strongly reflects the less compressed lattice in the dense phase. Furthermore, the doublet peaks observed at 116 and 138 cm^–1^ are related to intermolecular interactions.^29^ These vibrations are associated with the C–C stretching vibration of the alkyl chain in the propionic acid and methylpropyl groups. The difference in the peak intensity and peak broadening in the region of 100–270 cm^–1^ is related to CH_3_ torsional vibration and C–C deformation. On the higher-frequency side, three vibrations related to C–OH bending and deformation are observed in the region of 430–600 cm^–1^. These vibrations are highly sensitive to the environment, and thus, the solids show peaks at 478, 524, and 584 cm^–1^, whereas these weak vibrations are attenuated or absent for the dense phase. The region around 270–400 cm^–1^ is related to aliphatic C–C deformation mostly on the methylpropyl side. The changes in this region further confirm that the vibrational difference is not solely associated with dimer formation but also due to other intermolecular interactions in the dense phase. The interproton distance between the dense phase of the aromatic and aliphatic groups has been compared with crystalline solids using ^H^NMR spectra, and the results showed that the intermolecular distance in both phases is similar,^13^ which further corroborates the close packing in the dense liquid phase.

### 3.2 SEM Images of Pure and Additive-Added IBU

It was very challenging for us to direct the IBU to undergo LLPS followed by core–shell structure formation as there is no standard methodology to follow. Any additives that have a stronger hydrogen bond donating ability than the O–H (dimer) group could interact with the drug and thereby could direct and control the drug–drug interaction to produce IBU crystals with a complex morphology. The additives were hydroxypropyl methylcellulose (HPMC), Pluronic-F127, polyvinylpyrrolidone (PVP), polyvinyl alcohol (PVA), and polyacrylic acid (PAA), and their amounts were in the range of 0.3–5 wt %. SEM images of HPMC (hereafter, IBU-HPMC-Precipitate)- and Pluronic-F127 (IBU-Pluronic-Precipitate)-assisted IBU crystallization by precipitation via cooling and single crystals via slow evaporation (IBU-HPMC-Slow evaporation, IBU-Pluronic-Slow evaporation) in the binary solution of 2-propanol and water are presented in Figure S6 and discussed in Section S3.3 (Supporting Information). Compared with Pluronic-F127, the addition of HPMC suppresses the XRD peak intensity related to the (100), (200), and (202) planes that hold the dimethyl group and carboxylic acid (Figure S7a,b, Supporting Information). In short, these two additives merely change the morphology but do not control the drug to produce a core–shell structure.

**Figure 2:**
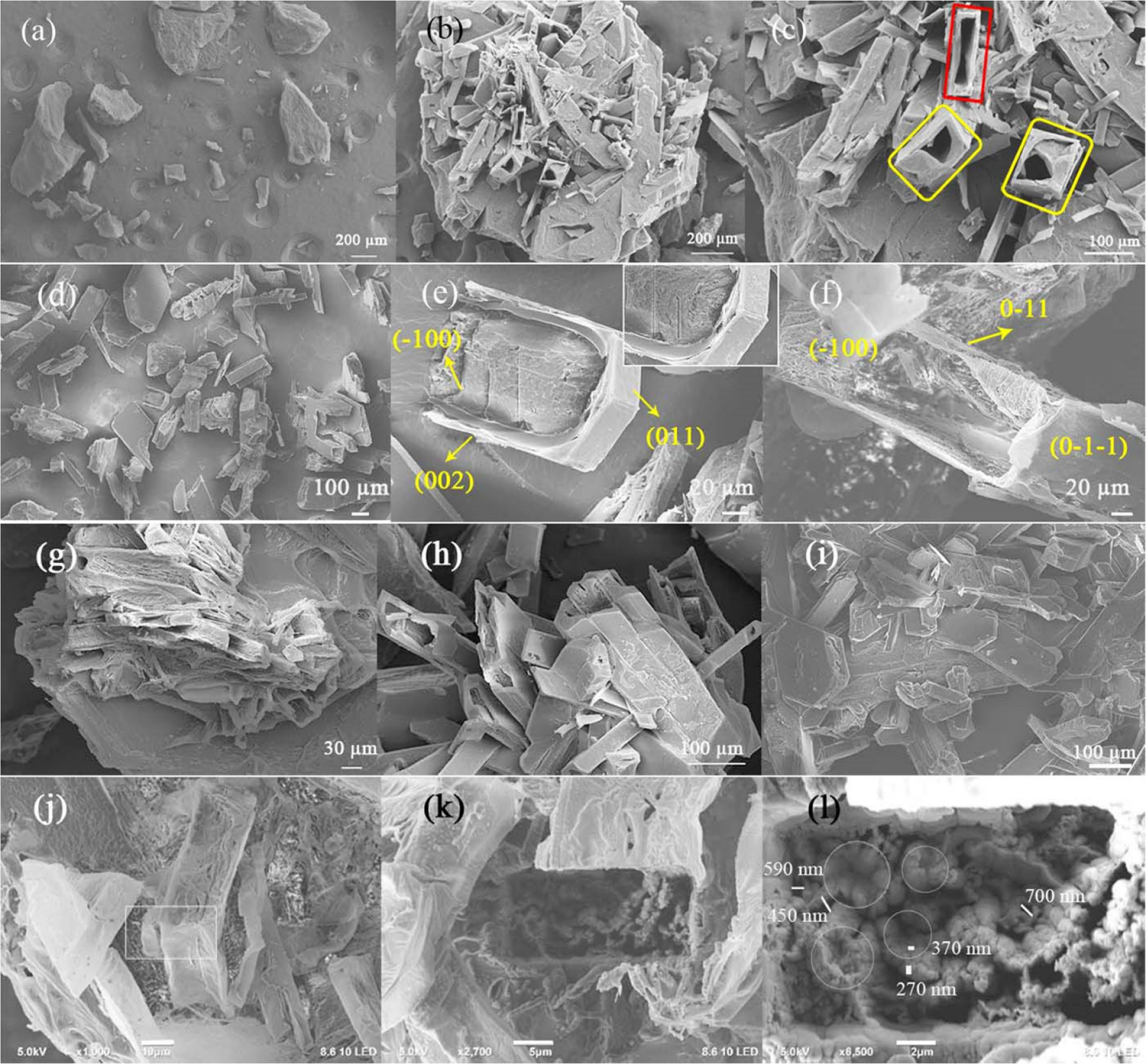
SEM image of the IBU 0.3 wt % PAA precipitate (a). Core–shell structure of IBU with 0.3 wt % PAA obtained by slow evaporation (b) and a magnified view (c). The partially dissolved core with a faceted single-crystal wall (yellow rectangle). The red rectangle highlights the core–shell structure. SEM images of IBU 0.3 wt % PAA with a core–shell morphology obtained by slow evaporation (d–f). Unstable nanoparticle aggregates at the hexagon core and the outer single-crystal wall are clearly presented I. IBU crystals with (g) 0.75, (h) 1.5, and (i) 5 wt % PAA. No core–shell structure was observed on increasing the PAA amount. The IBU PAA crystals with 0.3 wt % before (j) and after (k) cutting by FIB. The magnified view (2 μm length scale) of the internal structure (l) confirms the attachment (a periodic array of circles) of nanoparticle building units and voids between them inside the core.

The precipitate of IBU without the addition of additives and with PVP and PVA is presented in Figure S8a-i (Supporting Information). Compared with HPMC, Pluronic-F127, and PVP polymers (Figure S6), and the limited number of donor and acceptor additives (PVA) in slow evaporation produce crystals instead of a thick and stable solution of IBU. This has been taken as evidence to investigate one more additive, PAA, in IBU crystallization with different solvent ratios. PAA has a COOH group similar to IBU, which can form hydrogen bonding with IBU. This means that not only the additive interaction with the drug but the drug can also disturb the polymer hydrogen-bonding interaction.^30^ The precipitate with 0.3 wt % PAA with pH = 5.06 (Figure S9 Supporting Information) and without pH did not produce a core–shell morphology (Figure 2a). Surprisingly, when the solvent ratio was 1.5% with PAA addition of 0.3%, a spectacular core–shell structure with a mixture of rectangular and square outer cross-sections was formed after the LLPS (Figure 2b,c). The crystals were rectangular or square rods with a defined and deep hollow space. The internal wall of the crystals is not a well-faceted and roughened surface. In some crystals, the cavity is incomplete, and the material filling the space is rough. Notably, PAA is a better growth controller and produces a core–shell morphology compared with other additives.

In Figure 2d–f, the crystalline outer walls are well faceted, and the inner side contains porous and rough surfaces of nanoparticle aggregates. These aggregates may dissolve from inside to outside and finally produce a core–shell morphology. In another way, multiple nucleations may initially occur on the surface of the polycrystalline core, and crystallization extends from the surface to core by following reversed crystal growth via NCC.

To further understand the core–shell structure formation, we increased the amount of additive to 0.75, 1.5, and 5 wt % PAA (Figure 2g–i). A high amount of additives inhibited the core–shell structure formation as proven by 5 wt % PAA, which produced only single crystals, as shown in Figure 2i.

As an equal amount of water and 2-propanol produce a very stable LLPS instead of crystals at a solvent ratio of 1.5%; it is obvious that the water has played a greater role in the interaction with IBU. This creates further interest to investigate the effect of water and ethanol as a binary solvent. Irrespective of the presence of PAA and its amounts, rectangular plate crystals were observed, as presented in Figure S11 (Supporting Information). In other words, the (100) face is dominant, which is consistent with the ethanol-grown IBU.^31^ Unlike water/2-propanol, LLPS is not observed for water/ethanol. The crystal preserves its inherent isometric prism morphology as there is no significant change in the dominant peaks; however, some planes such as (300), (102), and (202) show a strong change in the intensity, and the addition amount of PAA consequently suppresses these peak intensities (Figure S12, Supporting Information).

### 3.3 SEM-Interfaced Focused Ion Beam (FIB) Cutting

Although the core–shell structure is obvious, it is important to visualize the cross-section of the core as there is a deceptive relationship between single crystals and crystals grown via nonclassical crystallization.^32^ A cross-sectional view of crystals shows the building unit attachments inside the crystals, so we performed an FIB cutting to confirm the internal arrangements (Figure 2 j–l). The size range of the building units at the core is 250–350 nm. These nanoparticles are oriented and attached together randomly. It creates the domain of attached particles, as circled in Figure 2l, with void sizes in the range of 450–800 nm.

### 3.4 Mechanism of Dense-Phase Transformation and Core–Shell Structure Formation

Before discussing the mechanism, we briefly describe the already suggested mechanism for the transformation of the amorphous liquid phase to a crystalline hierarchical structure. Several research reports have shown the transformation of liquid precursors to hierarchical mesostructures.^33^ In this well-studied report on the multistep crystallization of dl-glutamic acid, the polymer-induced liquid precursor (PILP) forms first under the control of oppositely charged polyethylene imine, and subsequently, the homogeneous nucleation of nanoplates occurs within the droplet as a second step. Finally, a reorientation of platelets creates a hierarchical microsphere. Similarly, indomethacin microspheres on the amorphous precursors followed two different pathways to produce a hierarchical structure.^34^ To advance these results, the direct observation of the nanoscopic precursor using liquid-phase AFM and attachment of particles for the further growth via NCC has been reported.^6^ This study offers insights into previously unrecognized handles for the transformation of the crystalline phase from liquid precursors. Nonetheless, the authors used different terminologies such as PILP, although it should be noted that the experimental steps they followed to facilitate LLPS were similar, e.g., cooling to room temperature from 60 °C and use of polymers.

**Scheme 2:**
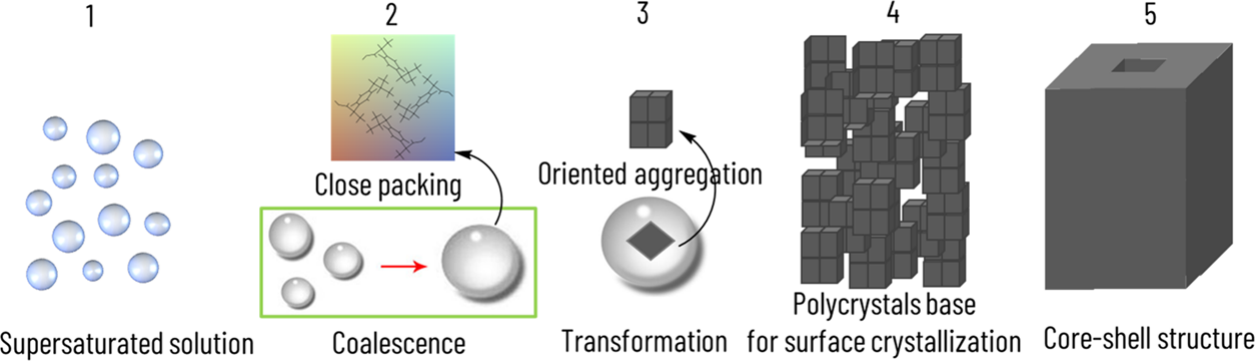
Schematic Representation of LLPS Formation and its Transformation to a Core–Shell Structure in NCC LLPS was formed (1); then, the dense liquid phase coalesced into large droplets (2) (also refer Scheme 1 SI); the Raman studies confirmed that the inter- and intramolecular interactions occurring in the dense phase were similar to those in a close-packed crystal structure (3); nucleation was discerned in the dense phase (3), after which the aggregation of nanocrystals produced the mesostructure (4); finally, dissolution and recrystallization of the metastable core into a crystalline outer surface occurred (5).

Although we used different water/2-propanol compositions such as 1.5, 1, and 4, LLPS, followed by a core–shell structure, was obtained for the ratio of 1.5. A solvent ratio of 1 produces a very stable and glassy-like amorphous phase as the end-product. Similar phenomena of a stable amorphous phase have been observed for idebenone.^35^ As the amount of solute dissolved has a direct relationship with the amount of 2-propanol, a solvent ratio of 1 produces stable glassy amorphous materials.^36^ In our case, the low melting point (78.8 °C, Figure S13, Supporting Information), solvent selection and their composition, additives, and rapid cooling from 50 to 25 °C are the reasons for the formation of LLPS. The solute–solvent interaction must be considered to understand why LLPS occurred for the water/2-propanol solution but not for the water/ethanol and water/acetone solutions with a solvent ratio of 1.5. The polar–polar interaction between the COOH group of IBU and the OH group of 2-propanol and ethanol, the nonpolar–nonpolar interaction between the hydrophobic group of IBU and the alkyl chain of 2-propanol, and the interaction of ethanol and acetone should be considered. The two-alkyl chain of 2-propanol in the nonpolar interaction and OH interaction with IBU might be dominant to delay the nucleation compared with other solvents.^37^ A separate study combined with DFT is needed to derive a comprehensive understanding of solvent effects on the core–shell structure formation, which is not within the scope of this study (Scheme 2).

The transformation from LLPS to a core–shell morphology is completely new, which proves the role of LLPS in NCC. The similar molecular interactions both in the dense phase and in the crystalline solid confirm the structural ordering in LLPS, and thus it is considered as the precursor phase. A similar possibility of close packing was confirmed through *in-situ* H NMR studies.^13^ Nonetheless, IBU is a less hydrophilic molecule due to its inherently low polarity, which results in a lack of anchoring sites required for further organization; this dense phase with more solute may facilitate the close packing and so the structural ordering. As the solubility of the dense phase is high, metastable intermediates are possible due to high supersaturation. Later, close-packed molecules may assemble to produce nanoparticles. These nanoparticles can further orient in a crystallographic way or aggregate together to produce the core. Based on the stability or surface crystallization, it may further dissolve to produce a core–shell structure. In addition to SEM-FIB reported here, cryo TEM-SAED images may be required for further confirmation of the particle arrangement at the core,^38^ and the study is in progress. A similar core–shell morphology has been reported for the mesocrystallization of dl-alanine in water/isopropyl alcohol^39,40^ and reversed crystal growth of C-methylcalix[4]resorcinarene via NCC.^17^ When discussing the core–shell morphology, it is important to consider the impurity-induced ripening in which structurally similar impurities (in our case additives) can be entrapped at the core in the initial stage of crystallization and dissolved during growth, resulting in a core–shell structure.^41^ However, this ripening process with PAA may be ruled out in our case as we did not observe any core–shell morphology on adding a higher amount of PAA of 1.5 and 5 wt % (Figure 2g–i).

**Figure 3:**
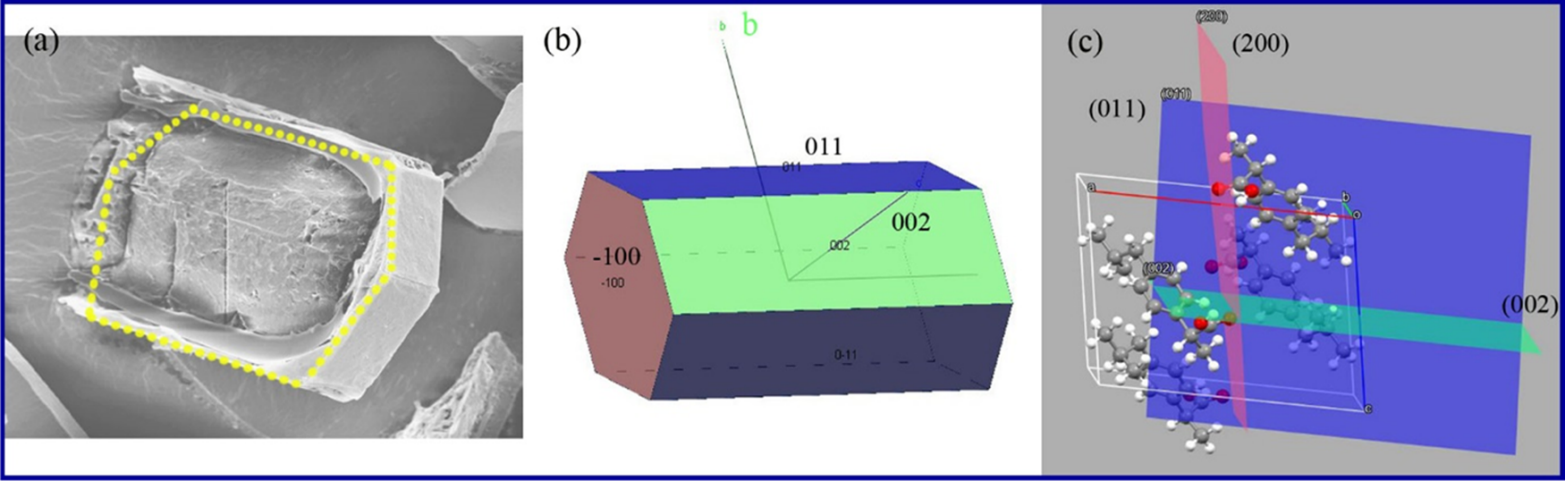
SEM image of IBU crystals with hexagon core (a) and the corresponding morphology generated using XRD data (b). The hydrophilic COOH group at the (200) plane is propagating perpendicular to the core (002) plane (c) which does not support the dissolution of hydrophilic group at the core to produce core–shell structure. The dotted yellow in (a) highlights the cross-sectional morphology shown in (b).

The core–shell structure was also reported for salicylic acid in which COOH is predominantly involved in the process.^42^ As water shows strong hydrogen bonding with COOH, propagating through the center,^43^ site dissolution occurred at the center and extended from inside to outside to produce a core–shell structure. This mechanism cannot be applied in our case because the COOH group in IBU propagates at 80.30° (an angle between the (200) and (002) planes) to the center along the (200) plane. The methyl in the methylpropyl group propagates through the center along the (002) plane, which may create an unstable phase due to van der Waals interactions between the phenyl ring and the aliphatic chains,^44^ resulting in dissolution from inside to outside. To further confirm, the cross-section of the morphology without the (002) plane is rectangular or square (Figure S10 see supporting information), which does not correspond to SEM images reported in Figures 3 or 2d–f, as the core is not dissolved completely for rectangle or square morphology. In addition to this, the probability of interaction with water is less as nucleation begins in the dense phase, dominated by the 2-propanol solution.

### 3.5 SAXS Experiment for the Liquid and Solid-Phase IBU

SAXS is a powerful technique to directly probe nucleation or low-resolution structures in solution. Simultaneous SAXS and WAXS has been successfully applied to investigate the nucleation and crystal growth of the organic small molecule 2,6-dibromo-4-nitroaniline.^45^ The study inferred that the nonscattering moieties or amorphous precursor phase formation is the first step before nucleation, which is then transformed to crystalline materials. A similar transformation has been uncovered via *cryo* TEM images.^4^

**Figure 4:**
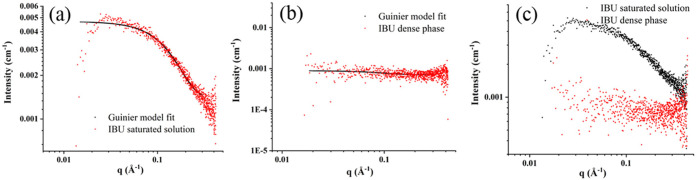
SAXS data of supersaturated solution (a) and dense phase (b) of IBU and their comparison (c).

**Figure 5:**
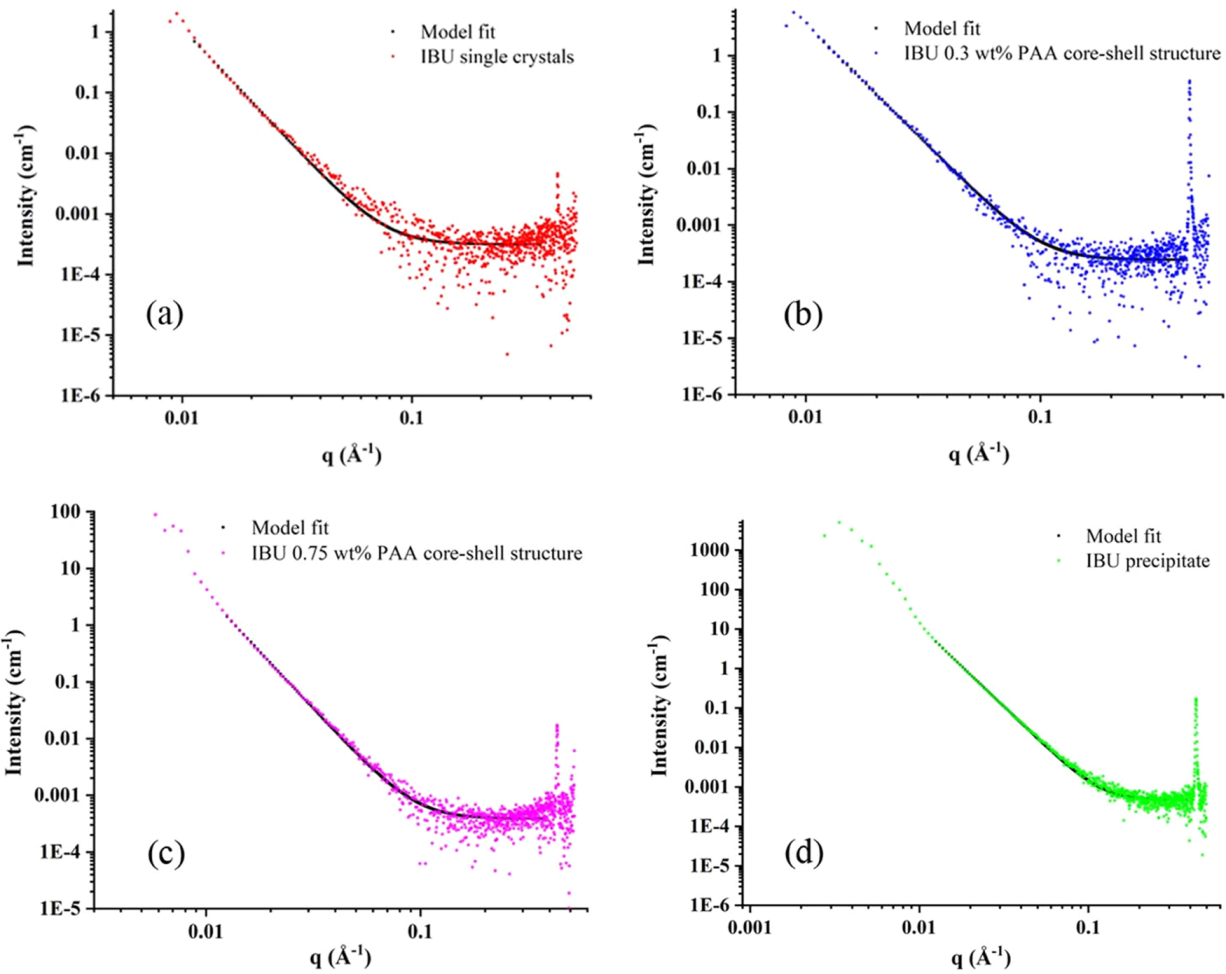
SAXS data of single crystals grown from a water/ethanol solution (a). IBU core–shell structure with 0.75 wt % PAA (b), with 1.5 wt % PAA (c), and IBU PAA precipitate (d). For all samples, the model was fit using the Guinier–Porod model.

In this study, SAXS data were collected for the supersaturated solution immediately after cooling (supersaturated solution), the dense phase (Figure 4), and the solid samples (Figure 5). Solid samples such as single crystals obtained from the water/ethanol mixture, IBU PAA precipitate, IBU core–shell structure with 0.75 wt % PAA, and that with 1.5 wt % PAA were also investigated. The R_g_ and Porod exponents for the different IBU samples are presented in Table S2 (Supporting Information). The fitted scattering data are presented in Table S2. The *R*_g_ value is 3.2 Å for the IBU monomer, while for the dimer, it is between 4 and 5 Å. When *R*_g_ equals 11.72 Å for the supersaturated solution, it indicates that the solution contains clusters of particles, as expected based on the cooling crystallization. The R_g_ value has doubled for the dense liquid phase, and the value is 18 Å, which is much larger than the dimer size of IBU. In Figure 4c, it is apparent that the scattering intensity has significantly decreased (21%) for the dense phase compared with the supersaturated solution, in other words, at low scattering density. As it is already a dense phase and the concentration does not impact the structural ordering, the decrease in intensity compared with the supersaturated solution is due to the interparticle interference effect, as described for monodisperse solutions of proteins.^46^ In this study, the horse myoglobin protein solution was approximated as spherical symmetric particles, and their size was calculated to be 4.7 nm. Furthermore, it must be taken into account that the absence of heavy elements or molecular moieties in IBU molecules does not boost the scattering intensity, which consequently makes it more difficult to detect very low levels of scattering density in the dense phase.

For the solid samples, the scattering from single crystals can differentiate other particles as it is highly ordered and has a crystallographic orientation. Strong differences in the scattering intensity were observed among the single crystals, core–shell structure, and precipitates (see Figure S14). Moreover, the influence of the crystal surface can affect the information associated with the internal structure.^47^ The scattering intensity is low for single crystals compared with both the core–shell structure and the precipitate, and the core–shell structure intensity is low compared with that of the precipitate. For single crystals, the density contrast at the scattering point is expected to be the same as the particles are large and thin plate-like crystals. However, in the case of the core–shell structure and precipitates, the particle size is small, and thus, there may be more electron density at the scattering boundary, resulting in high *R*_g_. The *R*_g_ value is high for the precipitate (*R*_g_ = 373), while the value is almost the same for the crystals and the core–shell structure (*R*_g_ = 140–190).

### 3.6 Dissolution Studies: Intrinsic Dissolution

**Figure 6:**
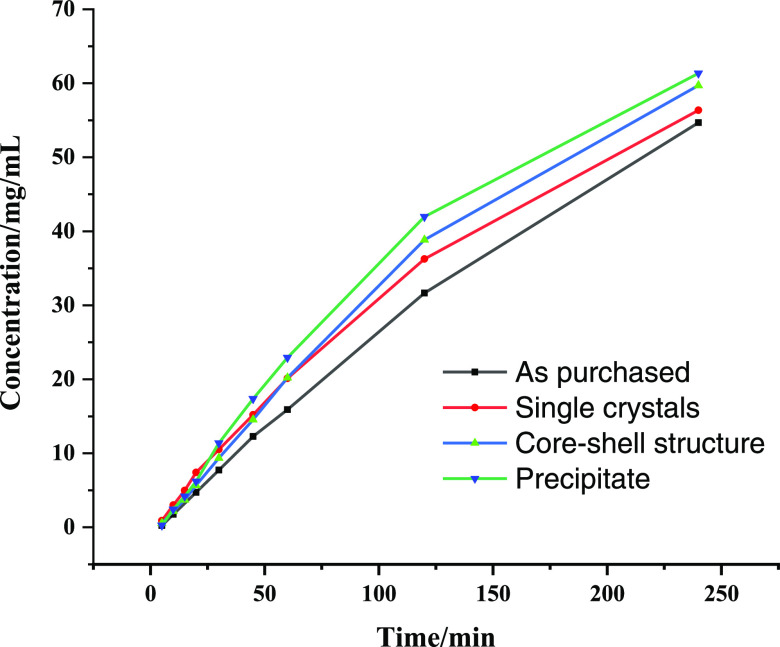
Dissolution profile of as-purchased, single crystals, core–shell structure, and precipitate of IBU.

The intrinsic dissolution behaviors of as-purchased, single crystals obtained from a water/ethanol solution, the core–shell structure, and precipitates are presented in Figure 6. Compared with as-purchased single crystals, all other samples show better dissolution behavior. There is no significant change in the drug release among the samples at the beginning; however, the difference is high around 120 mins. The precipitate shows high drug release compared with other samples due to the agglomeration of small nanoparticles. When the core–shell structure is exposed to a buffer medium, the internal structure alleviates the disintegration of the core easily, and it is thereby expected to have high dissolution. The drug release of the core–shell structure is higher than both the as-purchased and single crystals. Nonetheless, the core–shell structure shows higher dissolution compared with as-purchased single crystals, and the difference is about 7%. The presence of single crystals on the outer wall in the core–shell structure possibly reduces the dissolution rate. The preparation of a uniform core–shell structure may exhibit the true impact of dissolution.

## 4 Conclusions

The liquid–liquid phase separation (LLPS), followed by core–shell structure formation, confirmed the nonclassical pathway (NCC) of IBU crystallization. The dense phase formed has been identified as the intermediate precursor phase, and the liquid-phase Raman and SAXS results showed the molecular interaction and cluster size in the dense phase. Rapid cooling from 50 to 25 °C of the IBU solution followed by slow evaporation produced a core–shell structure through LLPS. From the Raman spectra, in addition to O–H bending and C=O stretching vibrations associated with dimer formation, a red shift (16 cm^–1^) at 3036 cm^–1^ related to the chiral center, a homogeneous red shift of the solid sample, and doublet peaks at 116 and 138 cm^–1^ associated with the C–C stretching vibration of the alkyl chain in propionic acid and the methylpropyl group, respectively, confirmed the intermolecular interactions in the dense phase to be similar to those of crystalline solids. The radius of gyration (*R*_g_) of the dense phase is almost one-third of the supersaturated solution, and the reduced scattering intensity by about 20% due to the interparticle interference effect further confirmed the close packing of the molecules. PAA assisted the core–shell structure formation compared with other additives such as HPMC, Pluronic-F127, PVP, and PVA. PAA additionally influences the (102) plane, which passes through the alkyl chain in propionic acid and the methylpropyl group along the (202) plane. Based on the SEM-FIB, Raman, and SAXS results, the mechanism hypothesized is that after LLPS formation, the high supersaturation at the dense phase produced a polycrystalline core or an oriented assembly. This core was formed by weak van der Waals interactions between the phenyl ring and aliphatic chains. Later, a transition occurred such that dissolution took place at the core to produce the core–shell structure. The mechanism is based on reversed crystal growth or mesocrystal formation in existing reports; however, efforts are underway to identify the assembly of the core using *cryo* TEM images and SAED patterns. The dissolution of the core–shell structure was enhanced compared with as-purchased and single crystals of IBU.

## Supporting Information Available

Experimental flow-preparation method; image of liquid–liquid phase separation; details of experimental characterizations by X-ray powder diffraction, Raman spectra of liquid and solid samples, SEM and SEM-FIM, TG//STA, small-angle X-ray scattering, and dissolution test; results and discussion of powder X-ray diffraction; Raman spectra of dense and solid phases; SEM images of the influence of additives on IBU; X-ray powder diffraction of IBU precipitate with HPMC and Pluronic-F127 additives; packing diagram of IBU along the (110) and (202) planes; SEM images of IBU with PVP and PVA additives, IBU precipitate with PAA, IBU core–shell structure, and IBU obtained from a binary mixture of water and ethanol; powder X-ray diffraction pattern of PAA-added IBU; packing diagram of IBU along the (300), (202), and (102) planes; TG/STA plot of the IBU core–shell; Rg and Porod exponent of IBU; comparative plot of SAXS (PDF)
