# Supplementary material for: Nonclassical Crystallization and Core–Shell Structure Formation of Ibuprofen from Binary Solvent Solutions
Source: Cryst Growth Des. 2022 Dec 21;23(1):236–45. doi: 10.1021/acs.cgd.2c00971 (PMC9817074; doi:10.1021/acs.cgd.2c00971)
Supplement: Supplementary file 1 — cg2c00971_si_001.pdf [file cg2c00971_si_001.pdf]

Nonclassical Crystallization and Core-Shell Structure Formation of Ibuprofen from Binary Solvent Solutions

Rajaboopathi Mani[1,2*], Leena Peltonen[3], Clare J. Strachan[3], Maarit Karppinen[4] and Marjatta Louhi-Kultanen[1*]

[1]Department of Chemical and Metallurgical Engineering, Aalto University, FI-00076 Aalto (Espoo), Finland

[2]Department of Physics & Nanotechnology, SRM Institute of Science & Technology, Kattankulathur- 603203, Tamilnadu, India

[3]Drug Research Program, Division of Pharmaceutical Chemistry and Technology, University of Helsinki, 00014 Helsinki, Finland

[4]Department of Chemistry and Materials Science, Aalto University, FI-00076 Aalto (Espoo), Finland

Emails: marjatta.louhi-kultanen@aalto.fi and mrajaboopathi@gmail.com

Supporting Information

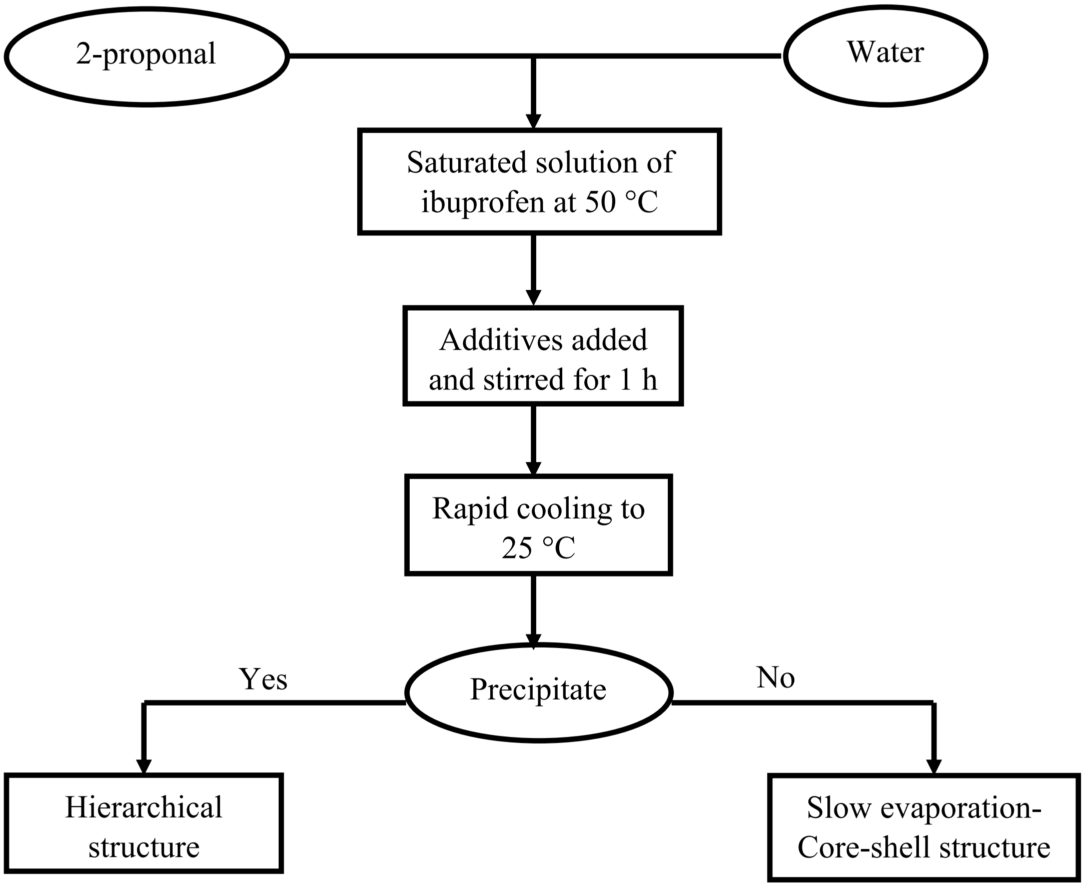

Fig. S1. Experimental flow of preparation of ibuprofen precipitate and core-shell structure.

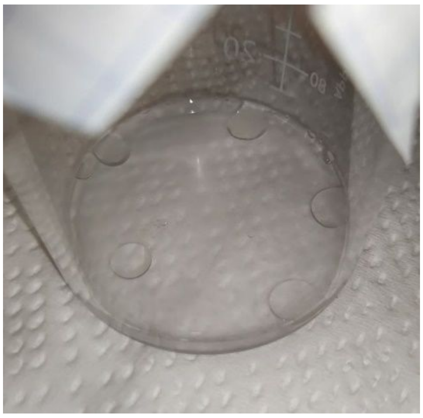

Fig. S2. Image of liquid-liquid phase separation (LLPS) recorded during crystallization.

S1. Characterizations

S1.1. X-Ray powder diffraction

The X-ray powder diffraction patterns were recorded on a PANalytical X'Pert PRO MPD Alpha 1 using Cu Kα radiation (λ = 1.5406 Å) at 45kV over the 2θ range of 5-60° with step size 0.02° and 20 s per step. The KrystalShaper (Version 1.5.0) was used to create the crystals morphology.

S1.2. Raman spectra of dense liquid and solid samples

Renishaw inVia™ confocal Raman microscope was used to record the vibrational spectra for the IBU dense phase and IBU precipitate. The dense phase spectra were compared with the solid to investigate the structural order in the former case. An emission wavelength of 785 nm (nIR) was selected as an excitation source in a continuous-wave gas laser. The dense phase in LLPS was carefully collected and placed under the microscope and the objective lens (x63) of Leica imaging microscope probe was immersed to collect the spectra. A noncontact objective lens 20x was focused and spotted the crystalline samples to record the solid spectra. The spectra were collected in the region 100-3200 cm^{-1} with 10 s exposure time and three accumulations. The 830 lines/mm grating was used to collect the whole spectra region and 1800 lines/mm grating was used to collect the mid and low-frequency region. The Wire 5.3 software was used to collect data.

S1.3. Scanning electron microscope

The morphology of the samples was examined using Zeiss Sigma VP scanning electron microscope (SEM) with an accelerating voltage 5 kV and InLens detector. The samples were fixed on a metal stud using two-sided carbon tape and the samples were sputtered approximately 10 nm using Au Pd using Leica EM AC600 sputtering device to avoid the conductivity issues. Image J software was used to analyze the particle size from SEM images.

S1.4. SEM interfaced focused ion beam (FIB)

The cross-sectional image of the core-shell structure was obtained using SEM interfaced focused ion beam experiment. The JEOL JIB-4700F was used with an accelerating voltage of 30 kV and Ga+ ions were employed to cut the core-shell structure. The particles were mounted on an aluminum stud and Au Pd was coated similar to SEM measurement. To get a smooth cut, the sample under vacuum was coated with a 1 µm layer of platinum. Different beams were used for fine and coarse milling of the samples. Since the angle between ion beam and to the SEM detector is 45°, the cut sample was tilted so, and their cross-sections were imaged.

S1.5. TGA/STA Thermal analysis

The simultaneous thermal analyzer NETZSCH STA 449 F3 Jupiter & QMS Aeolos Quadro was used to measure the mass change and thermal effect of the core-shell structure and precipitate. The temperature range was in the region 40-600 °C at a heating rate of 10 K/min in the crucible of Al_2O_3 85 µl under a flow of helium purge gas at the rate of 70 mL/min.

S1.6. Small angle X-ray scattering of liquid and solid samples

The different IBU liquid samples such as supersaturated solution after cooling and IBU dense phase and other solid samples (IBU single crystals and IBU precipitate) were investigated using

SAXS technique. A Xenoxs Xeuss 3.0 interfaced with Full travel in-vacuum motorized HPC detector was used. The sample to detector distance was 0.5 m and the X-ray wavelength was λ = 1.542 Å. For the liquid samples, the supersaturated solution immediately after cooling and dense phase collected from LLPS were separately filled in microtubes using micro syringe and both ends were sealed completely. The SAXS was performed for all the samples at a time. A SasView 5.0.4 software was used to fit the data. The SAXS data fit were performed by Guinier Porod model using following mathematical equation[1]. The model gives a good estimation of the particle size (radius of gyration, Rg) and diverse shape of the scattering particles (Porod exponent). For instance, the Porod exponent equal to one implies the rod-like shape of the particles.

$$I(Q) = G\exp\left(\frac{-Q^2 R_g{}^2}{3}\right) for\, Q \le Q1 \text{..........1}$$

$$I(Q) = \frac{D}{Q^4} for\, Q \ge Q1 \text{..........2}$$

$$Q_1 = \frac{1}{R_g}\left(\frac{3d}{2}\right)^{\frac{1}{2}} \text{..........3}$$

where I(Q) is the intensity, G Guinier factor, Q- difference between the incident and the scattered beam and Rg radius of gyration, Q1 transition point and d Porod exponent

For the dense phase, as the data does not fit well for the Guinier-Porod model, we choose Guinier model below to fit well in the low q region and this model is often used for monodispersed solution.

$$I(q) = scale.\exp\left[\frac{-Q^2 R_g^2}{3}\right] + background \text{..........4}$$

S1.7. Dissolution test

Dissolution test for the as purchased, single crystals, core-shell structure and precipitate of IBU was performed by following The United States Pharmacopoeia rotating disk method in phosphate buffer (pH 7.2, 900 mL) at 37 °C, using Distek dissolution system 2100B apparatus. The rotating speed was at 100 rpm and 100 mg of pelletized IBU was used for the dissolution. The drug sample was compressed at 1 ton for 90 s. At the fixed time intervals of 1, 2, 5, 10, 15, 20, 30, 45, 60, 120, 240, 360 min, the release medium of 5 mL sample was withdrawn, and an equal amount of fresh dissolution medium was added. Withdrawn samples were filtered through 0.45 µm membrane filter, and the concentration was quantified spectrophotometrically at the excitation of 221 nm wavelength using a UV-VIS spectrophotometer (Lambda 40, Perkin Elmer). The percentage of drug release versus time was plotted to realize the impact of the dissolution profile of different samples.

S2. Results and Discussion

S2.1. Powder X-ray diffraction

The X-ray powder diffraction patterns of as purchased racemic (±) IBU and IBU precipitate are presented in Fig. S3. The diffraction peak observed at the angle 2θ=6.12 (d=14.41) is the characteristic peak that differentiate the racemic (±) IBU from S(+)-IBU enantiomer. For the as purchased IBU, the growth along (110) direction is high, while for the IBU precipitate other planes such as (210), (211), and (112) are higher. There are no significant differences in peak positions or line shapes other than the intensity difference in the diffraction pattern.

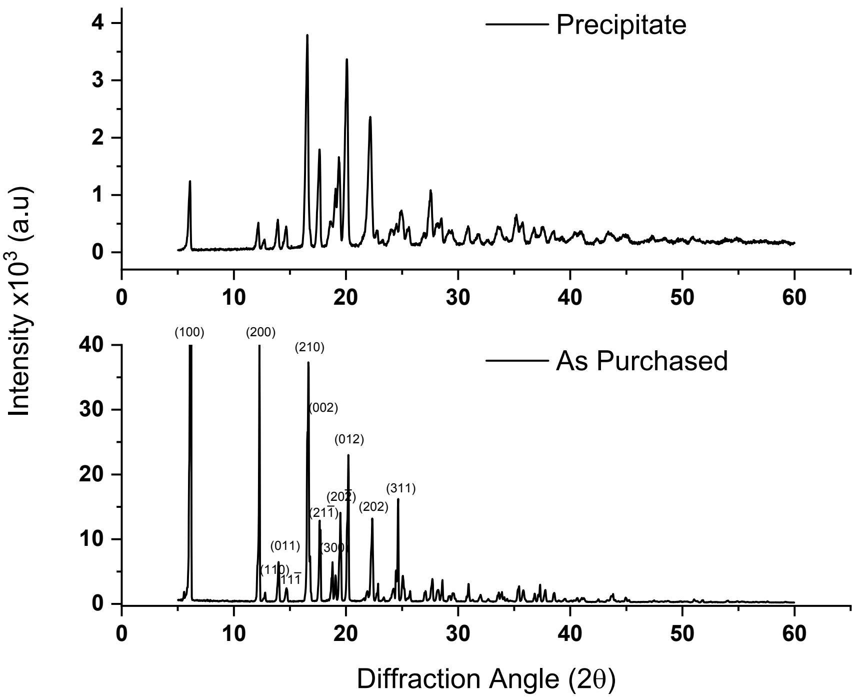

Fig. S3. Powder X-ray diffraction pattern of as purchased and precipitate of racemic (±) ibuprofen. As the peak intensity of (100) is significantly high and suppressing the height of other peaks, the intensity scale has been restricted to 40000 (y-axis) in the as purchased sample.

S2.2. Raman spectra of dense and solid phases

The Raman spectra of IBU in the range of 100-3200 cm^{-1} is presented in Fig. S4 and functional groups assigned for the vibrations are listed in Table S1. The molecular structure of IBU with atom numbering is presented in Fig. S5. The C-H vibrations are observed in the higher frequency region of 2800-3100 cm^{-1} and the vibrations associated with benzene (3059 cm^{-1}) have not shown any significant changes however major redshift (16 cm^{-1} and 3 cm^{-1}) respectively has been observed for the C-H associated to chiral center (3036 cm^{-1}) and CH_2 in methylpropyl (2869 cm^{-1}) for dense phase compared with the solid[2]. The redshift associated with CH_2 does not occur in hydrogen bond alone (dimer) compared with solvation shell elsewhere[3]. These red shifts due to methyl group that are not involved in hydrogen bonding support our claim that the vibrational changes are not only due to dimer formation in the IBU.

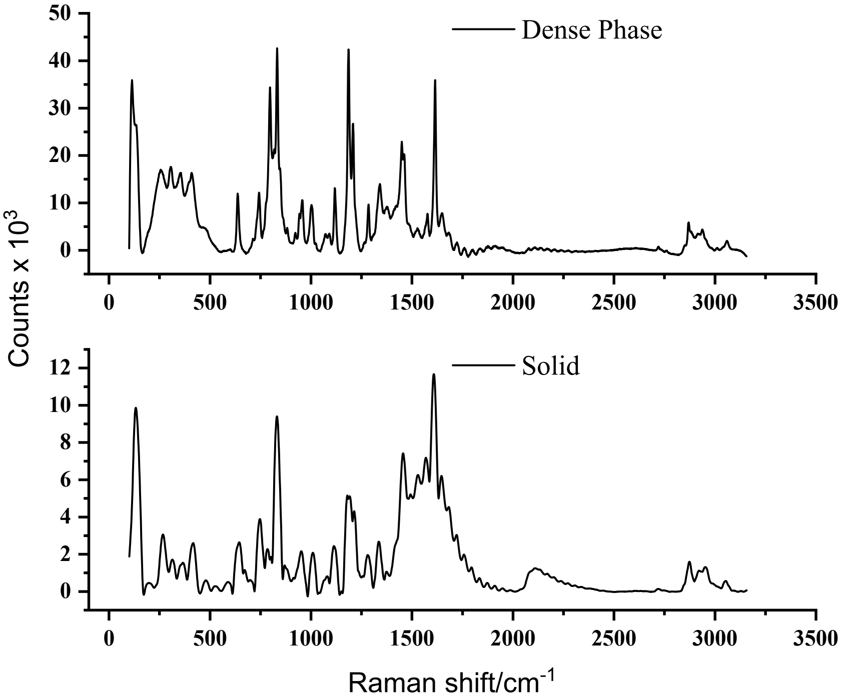

Fig. S4. The Raman spectrum of solid and dense phases. The grating used was 830 lines/mm.

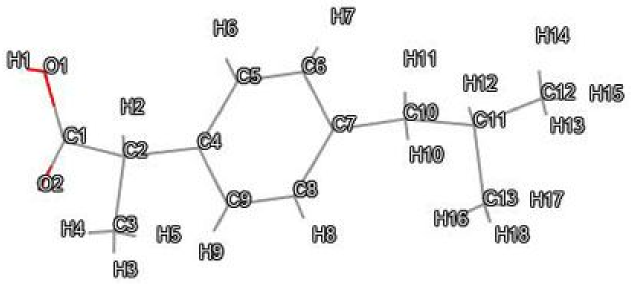

Fig.S5. Molecular structure of IBU (the atom numbering given only for vibrational analysis)

Table S1. The Raman vibrational assignments of IBU solid and dense phases.

S.No	IBU solid phase/cm^{-1}	IBU dense phase/cm^{-1}	Vibrational Assignments (refer Fig.S5 for atom numbering)
1	116	113	C7–C10–C11, C4–C2–C1 deformations
2	138	130	C7–C10–C11, C4–C2–C1 deformations
3	194	--	C3H_3–C, C13H_3–C torsions
4	222	--	C12H_3–C, C13H_3–C torsions
5	268	254	C12H_3–C, C13H_3–C torsions; C4–C2–C3 deformation
6	310	306	C10–C11–C12 deformation, C3H2–C4 deformation
7	327	--	C10–C11–C12 deformation

8	359	352	C10–C11–C13 deformation; C10–C2 wagging
9	384	--	C10 –C2 in-plane; out-of-phase bending
10	413	411	C12–C11–C13 deformations
11	478	481	C2–C1–OH deformation; ϕ in-plane bending
12	524	--	CO–H bending; out-of-plane bending
13	584	--	C2–C1–OH deformation
14	637	636	CO-H out of plane bending, in-plane bending
15	662	--	CO-H out of plane bending, C2-C1=O deformation, in-plane bending, in-pl. bend
16	691	--	C-OH stretching, C4-C2 stretching, out of-plane bending
17	746	742	CH_3 rocking, CH out of plane bending (ring vibration)
18	783	796	out of-plane bending, CH_3 rock; C=O out-of-plane wagging
19	819	817	$C3H_3$ rocking; C7–C10 stretch.; C11 (C10C12C13) sym. stretch.
20	833	832	CH out-of-plane bending
21	849	846	CH out-of-plane bending; $C3H_3$ rocking,
22	880	882	CH_2 rocking; $C13H_3$ rocking; C11H12 bending,
23	919	921	CH out-of-plane bend; $C12H_3$ rocking,
24	942	942	H8–C8C9–H9 out-of-phase bend; $C12H_3$ rock; $C13H_3$ rock, (CO–H bending (H-bonded))
25	950	--	H8–C8C9–H9 out-of-phase bend; $C12H_3$ rock; $C13H_3$ rock
26	958.58	957	$C3H_3$ rocking; C3–C2–C1 antisymmetric stretching
27	1007	1002	CH in-plane bend
28	1021		CH_2 twisting; $C3H_3$ rocking; C2–H2 bend,
29	1066	1071	CH_2 twisting; $C3H_3$ rocking; C2–H2 bend
30	1093	--	$C12H_3$ rocking; C11(C10C12C13) antisymmetric stretching,
31	1115.88	1117	CH in-plane bending
32	1181	1185	C7–C10 stretching

33	1207	1207	CH_2 twisting
34	1227	--	CO–H in-plane bending (H-bonded)
35	1285	1284	CH_2 wagging; C2–H2 bending
36	1340	1342	C11–H12 bending; C10–H10 bending,
37	1430	--	C11–H12 bending; C10–H10 bending,
38	1452	1448	$C12H_3$ and $C13H_3$ antisymmetric deformation,
39	1575	1577	C–C stretching
40	1608	1614	C–C stretching
41	1655	1652	Overtone C1=O2 stretching (H-bonded)
42	2872	2869	CH_2 symmetric stretching,
43	2952	2936	C2–H2 stretching,
44	3053	3059	H8–C8C9–H9 antisymmetric stretching,

S2.3. SEM images of HPMC and Pluronic, PVP and PVA influences on IBU.

The HPMC is a neutral polymer with abundant hydrogen bond donors and acceptors in the bulk structure (growth inhibitor). Similarly, Pluronic-F127 is copolymer which can be used as crystal size inhibitor. The solvent ratio used for the precipitate is 4 and 1.5 for the slow evaporation. The effects induced by HPMC and Pluronic-F127 are trivial towards the core-shell formation. The HPMC produces rough plate like and chunky particles with irregular shape. Since our aim is to control the particle formation at the initial stage of crystallization, but not to control the morphology, we used limited amount of additives for e.g. 0.3 wt % of HPMC (Fig.S6 a). The pH=4.83 environment has not impacted the morphology (Fig.S6 b) as it looks similar to Fig.S6a. The dense phase was harvested and dried before SEM measurement. The crystals are highly porous, and the long polymer chain has produced IBU in the form of fiber attached together. Similar to IBU-HPMC-Slow evaporation, the addition of Pluronic-F127 has not produced any core-shell structure. The pH (pH=5.35) environment along with Pluronic-F127 (Fig.S6e) produces aggregation which is different from addition of Pluronic-F127 alone as presented in Fig.S6d. The IBU-Pluronic-Slow evaporation (Fig.S6f) produces crystals similar as IBU-HPMC-Slow evaporation (Fig. S6c).

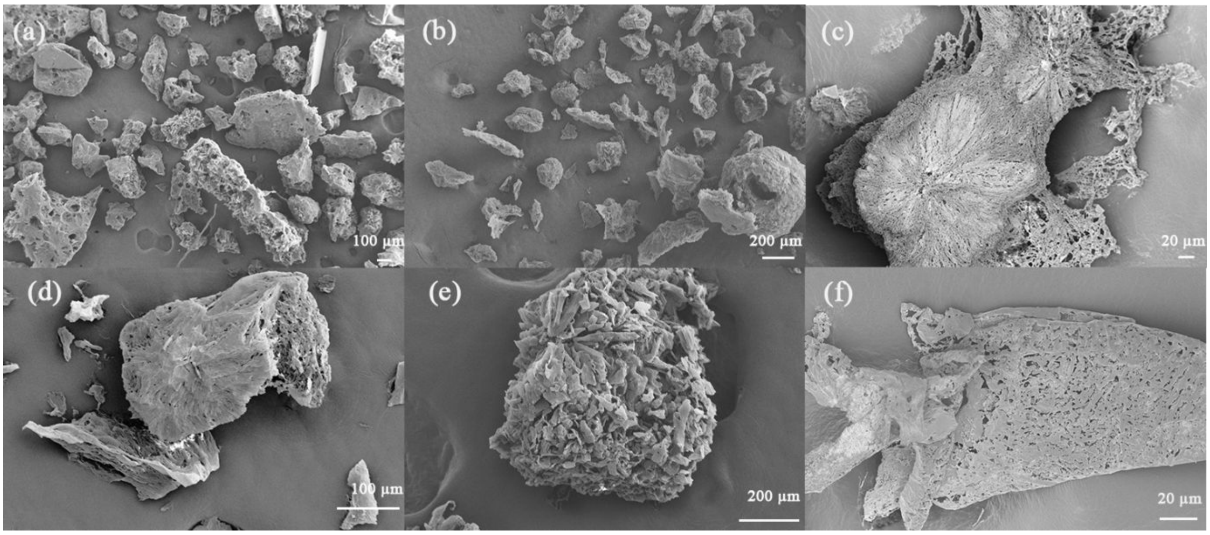

Fig S6. The SEM images of IBU prepared with addition of 0.3 wt % HPMC and Pluronic-F127. The water to 2-propanol ratios is 4 and 1.5. The solvent ratio 4 produces precipitates whereas 1.5 used for crystallization from slow solvent evaporation. (a) IBU HPMC precipitate and (b) IBU HPMC precipitate with pH=4.83 (c) IBU HPMC slow evaporation (d) IBU Pluronic precipitate (e) IBU Pluronic precipitate with pH=5.35 (f) IBU Pluronic slow evaporation.

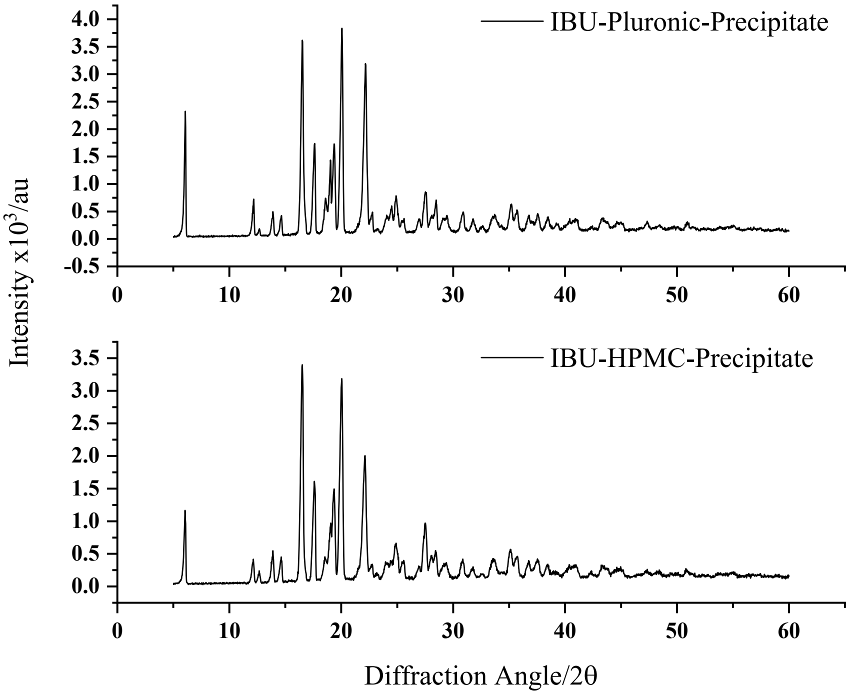

Fig. S7a. The powder X-ray diffraction pattern of IBU precipitate added *0.3 wt % HPMC and Pluronic-F127*.

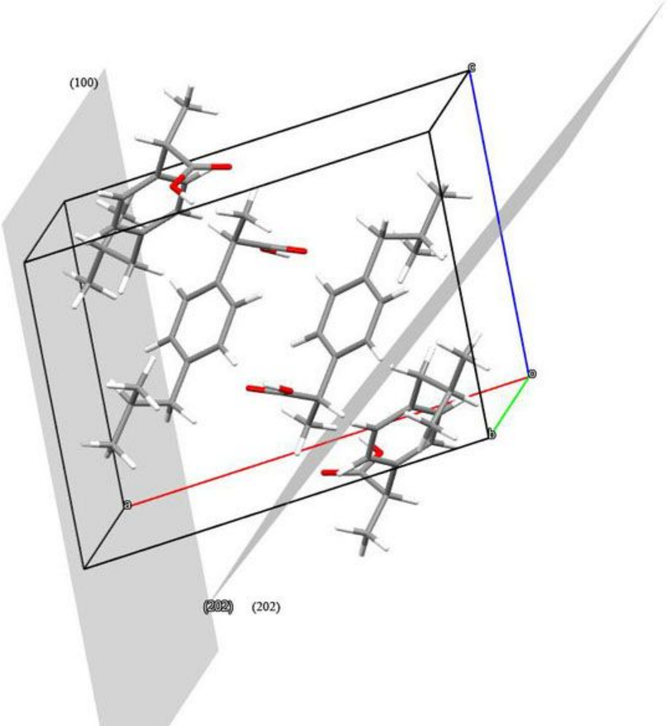

Fig. S7b. Packing of IBU and presence of (100) and (202) planes in the crystal structure. The HPMC and Pluronic additives influence these planes.

The PVP has a hydrogen bonding acceptor group without a donor. The PVA is amphiphilic and non-ionic polymer with hydrogen bonding donating and accepting groups whereas in PAA, there is an acid group similar to IBU with the presence of a donor group double that of the acceptor. The precipitate of IBU without the addition of additives and with PVP and PVA is presented in Fig. S8 (a-i). The precipitate shows chunky and rod-like morphology with a rough surface. The final products from the slow evaporation are porous and the magnified view shows the texture like structure (Fig.S8 (b-c)). The use of 1.5 wt % of PVP without pH and with pH=6.3 does not show any difference (Fig.S8 (d-e)) compared to precipitate of IBU without and with HPMC and Pluronic-F127 (Fig. S6). However, slow evaporation of 1.5 wt % PVP added IBU solution has grown crystals with clear facets in 3 days (Fig. S8f). The formation of core-shell morphology is absent in the case of precipitation and it was further confirmed by raising the PVA amount to 0.3 wt % without pH (Fig. S8g) and with pH= 5.08 (Fig. S8h). The slow evaporation of 1.5 wt % PVA added IBU solution with pH=6.25 has grown chunky crystals (Fig. S8i) without clear facets as shown in Fig.S8f.

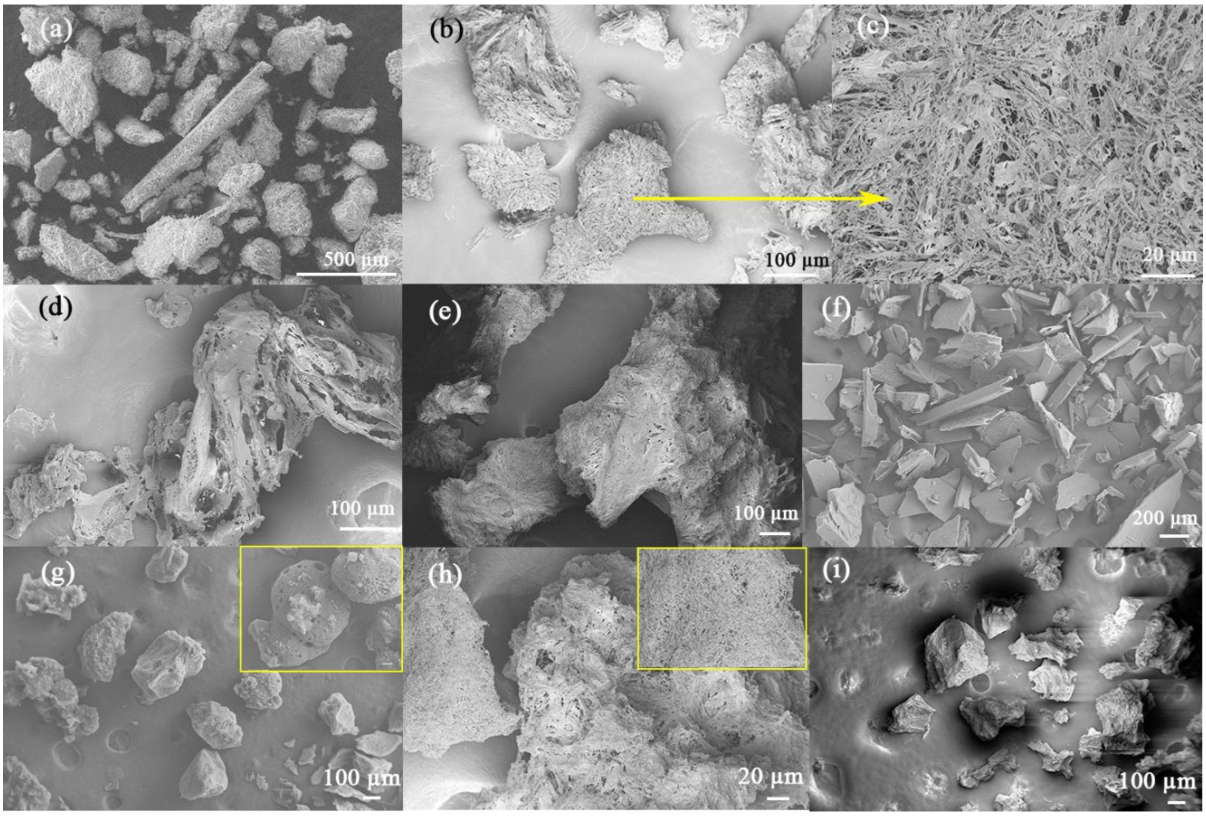

Fig. S8. The SEM images of IBU prepared without and with addition of Polyvinylpyrrolidone (PVP), Polyvinyl alcohol (PVA). The water to 2-propanol ratios were 4 and 1.5. The solvent ratio 4 produces precipitate whereas 1.5 used for crystallization from slow solvent evaporation. (a) IBU precipitate (b) IBU crystals from slow evaporation at pH 4.74 (c) magnified view (d) IBU PVP precipitate with 1.5 wt% (e) IBU PVP precipitate with 1.5 wt% at pH 6.3 (f) IBU PVP crystals with 1.5 wt% at pH 4.9 from slow evaporation (g) IBU PVA precipitate with 0.3 wt% (h) IBU PVA precipitate with 0.3 wt% at pH 5.08 (i) IBU PVA precipitate with 1.5 wt% at pH 6.25.

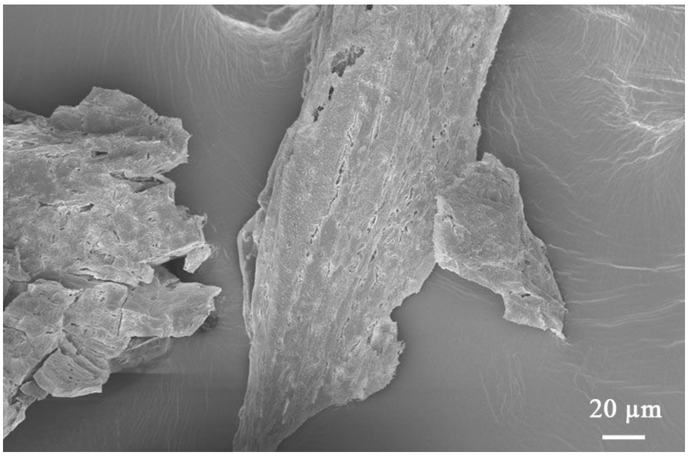

Fig. S9. The SEM image of IBU 0.3 wt % PAA precipitate with pH 5.06.

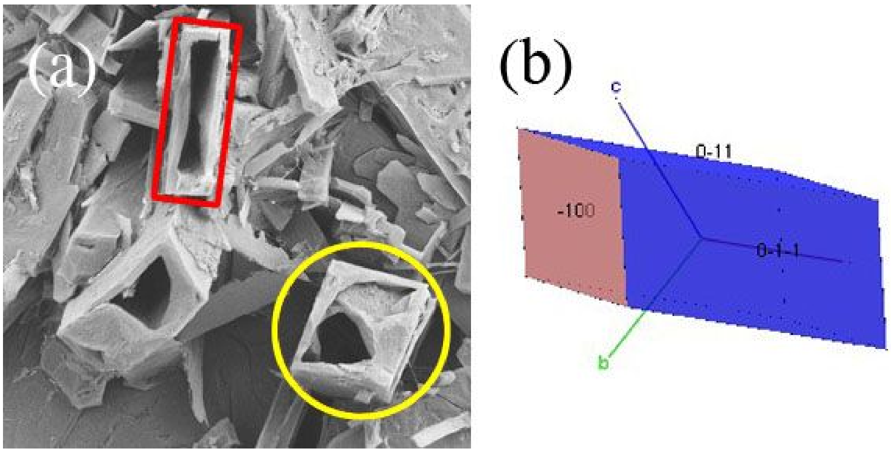

Fig.S10. The SEM image of IBU with 0.3 wt % PAA core-shell structure and corresponding morphology.

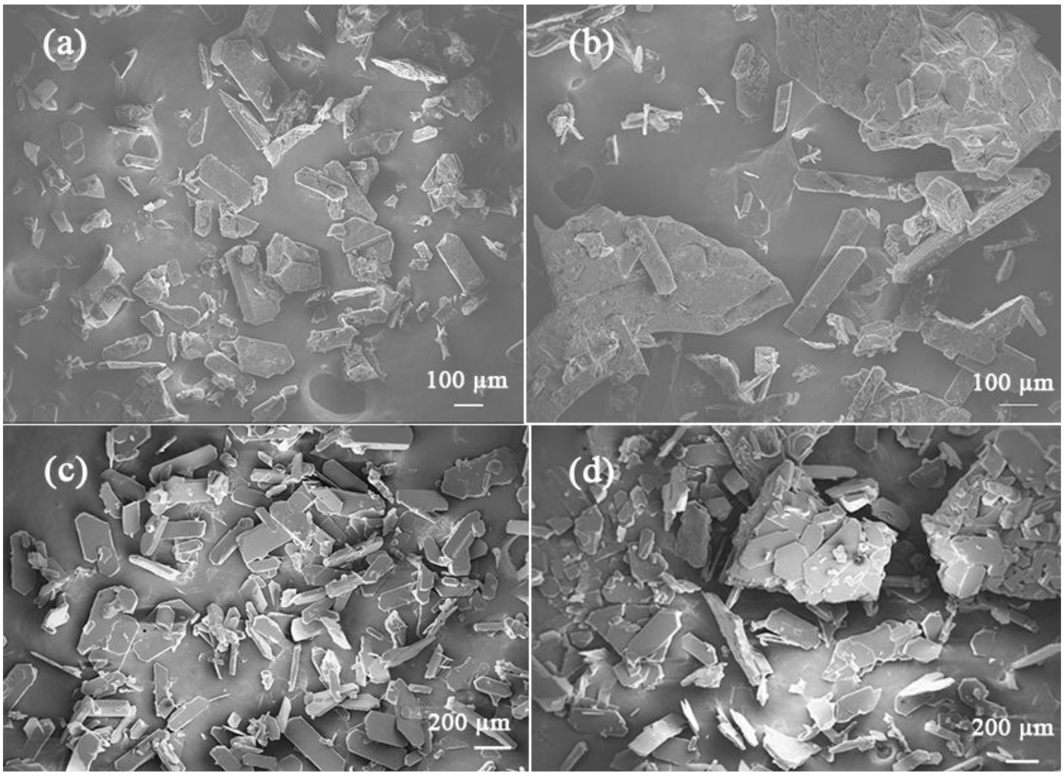

Fig. S11. The SEM image of crystals obtained in equal amount of water and ethanol. Crystals without additives (a), IBU with 0.3 wt% PAA (b), IBU with 1.5 wt% PAA (c), IBU with 5 wt% PAA (d).

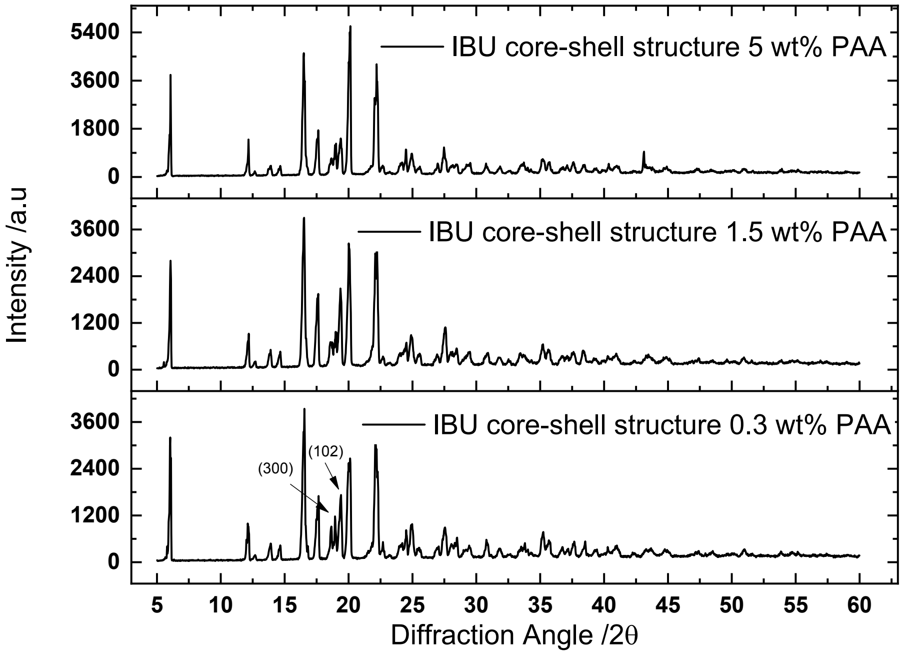

Fig. S12a. Powder X-ray diffraction pattern of polyacrylic acid (PAA) added IBU. The amount of PAA was 0.3, 1.5 and 5 wt % (bottom to top).

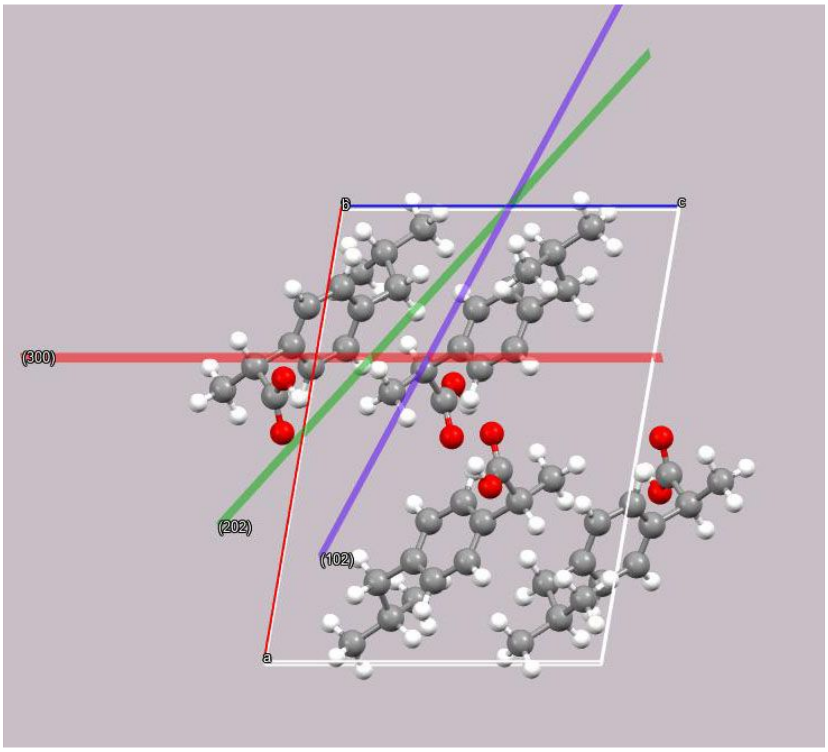

Fig. S12b. *Packing of IBU and presence of (300), (202) and (102) indices in the crystal structure. The PAA additives influence these indices. The angle between 202 and 102 is 13.92°.*

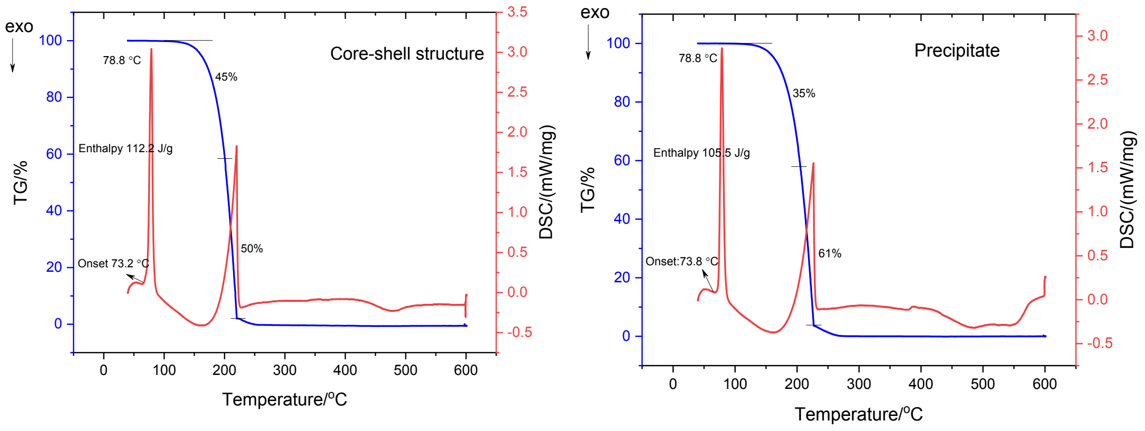

Fig S13. The TG/STA plot of IBU core-shell structure *and pure precipitate. There is no change in the melting point however, the enthalpy changes are observed between the two samples. The percentage of mass loss occurs at different stages for the two samples.*

Table.S2. The Rg and Porod exponent values obtained from Guinier and Guinier-Porod model for the different IBU samples.

S.No	Sample	Rg/Å	Porod Exponent	Fitting range
1	IBU supersaturated solution	11.72	--	0.015-0.291
2	IBU dense phase	18.00	--	0.020-0.336
3	IBU single crystals	139.05	4.01	0.011-0.363
4	IBU core-shell structure with 0.75 wt % of PAA	196.86	4.12	0.011-0.407
5	IBU core-shell structure with 1.5 wt % of PAA	186.14	4.03	0.012-0.374
6	IBU PAA precipitate	391.98	4.16	0.010-0.381

The Porod exponent is 3<P<4 for the crystals which implies the rough surface. For other solid samples, the value is greater than four, which means the particles are smooth or the particle have somewhat diffused boundary. In case of diffused boundary, the electron density profile in the scattering center is not same and thus shows density contrast between the two particles.

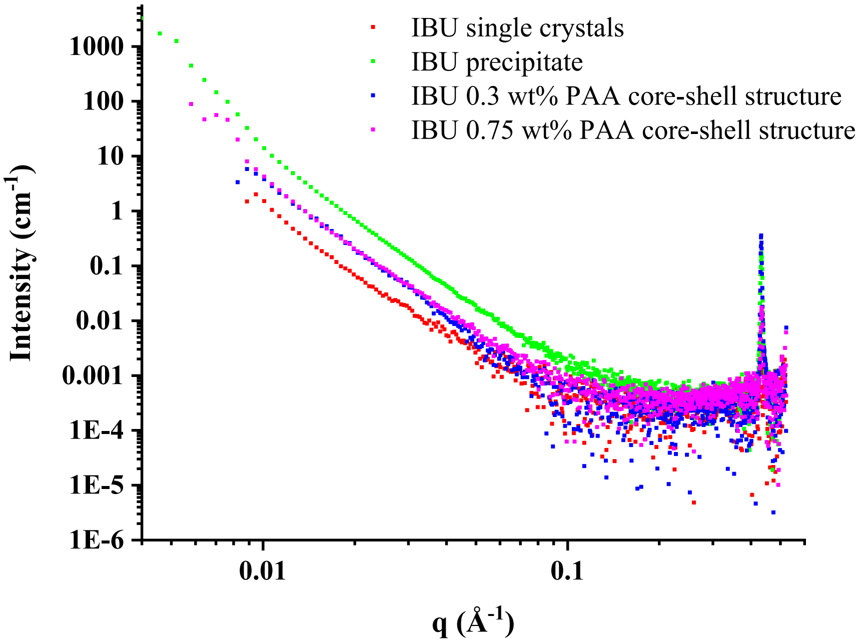

Fig. S14. The comparison plot of SAXS for the IBU PAA core-shell structure 0.75 wt % (b), IBU PAA core-shell structure 1.5 wt % (c) and IBU PAA precipitate (d).

References

(1) Hammouda, B. A New Guinier–Porod Model. *J Appl Crystallogr* **2010**, *43* (4), 716–719.

(2) Vueba, M. L.; Pina, M. E.; Batista De Carvalho, L. A. E. Conformational Stability of Ibuprofen: Assessed by DFT Calculations and Optical Vibrational Spectroscopy. *J. Pharm Sci.* **2008**, *97*, 845–859.

(3) Bondesson, L.; Mikkelsen, K. v.; Luo, Y.; Garberg, P.; Ågren, H. Hydrogen Bonding Effects on Infrared and Raman Spectra of Drug Molecules. *Spectrochim. Acta A Mol. Biomol. Spectrosc.* **2007**, *66*, 213–224.
